# Presynaptic Calcium Channel Open Probability and Changes in Calcium Influx Throughout the Action Potential Determined Using AP-Waveforms

**DOI:** 10.3389/fnsyn.2020.00017

**Published:** 2020-04-24

**Authors:** Matthew S. Scarnati, Stephen G. Clarke, Zhiping P. Pang, Kenneth G. Paradiso

**Affiliations:** ^1^Department of Cell Biology and Neuroscience, Rutgers University Piscataway, Piscataway, NJ, United States; ^2^Department of Neuroscience and Cell Biology and Child Health Institute of New Jersey, Robert Wood Johnson Medical School, New Brunswick, NJ, United States; ^3^Graduate Program in Biomedical Engineering, Rutgers University Piscataway, Piscataway, NJ, United States; ^4^Child Health Institute of New Jersey, Robert Wood Johnson Medical School, New Brunswick, NJ, United States

**Keywords:** action potential, calcium channels, local calcium concentration, nanodomains, microdomains, open probability, presynaptic, neurotransmitter release

## Abstract

Action potentials arriving at a nerve terminal activate voltage-gated calcium channels and set the electrical driving force for calcium entry which affects the amount and duration of neurotransmitter release. During propagation, the duration, amplitude, and shape of action potentials often changes. This affects calcium entry, and can cause large changes in neurotransmitter release. Here, we have used a series of amplitude and area matched stimuli to examine how the shape and amplitude of a stimulus affect calcium influx at a presynaptic nerve terminal in the mammalian brain. We identify fundamental differences in calcium entry following calcium channel activation by a standard voltage jump vs. an action potential-like stimulation. We also tested a series of action potential-like stimuli with the same amplitude, duration, and stimulus area, but differing in their rise and decay times. We find that a stimulus that matches the rise and decay times of a physiological action potential produces a calcium channel response that is optimized over a range of peak amplitudes. Next, we determined the relative number of calcium channels that are active at different times during an action potential, which is important in the context of how local calcium domains trigger neurotransmitter release. We find the depolarizing phase of an AP-like stimulus only opens ~20% of the maximum number of calcium channels that can be activated. Channels continue to activate during the falling phase of the action potential, with peak calcium channel activation occurring near 0 mV. Although less than 25% of calcium channels are active at the end of the action potential, these calcium channels will generate a larger local calcium concentration that will increase the release probability for nearby vesicles. Determining the change in open probability of presynaptic calcium channels, and taking into account how local calcium concentration also changes throughout the action potential are both necessary to fully understand how the action potential triggers neurotransmitter release.

## Introduction

Action potential amplitude, duration, and shape can change during repeated activity, with exposure to certain drugs or toxins, as a result of some pathologies, and simply during propagation. The duration, amplitude and the speed of the rising and falling phase of the action potential are controlled by the activation and density of voltage-gated sodium and potassium channels. In nerve terminals, the depolarizing phase of an arriving action potential initiates the activation of voltage-gated calcium channels, and this activation continues during the repolarization phase of the action potential (Bean, 2007). The electrical driving force for calcium entry steadily decreases during the depolarizing phase and steadily increases during the repolarization (Bischofberger et al., 2002). Since the triggering of neurotransmitter release is highly sensitive to the local presynaptic calcium level (Heidelberger et al., 1994; Augustine, 2001; Neher and Sakaba, 2008) the shape and duration of the action potential and the peak amplitude of the action potential are major determinants of calcium channel activation and calcium entry which determines the amount of synaptic vesicle release (Wu et al., 2004; Yang and Wang, 2006; Hoppa et al., 2014).

To study voltage-gated calcium channels, standard voltage jumps are typically used to determine the amount and rate of calcium channel activation and inactivation at different stimulus voltages. The calcium channel tail current that occurs when the membrane potential is hyperpolarized can be used to measure the number of calcium channels that are opened at different test potentials (Eckert and Ewald, 1983). Although step depolarizations are an effective and informative method for studying voltage-gated calcium channels, this type of stimulus is of course not physiological. Accordingly, several groups have used AP waveforms or AP-like stimuli to study calcium channel activity (McCobb and Beam, 1991; Borst and Sakmann, 1998; Pattillo et al., 1999; Sheng et al., 2012) and neurotransmitter release (Wu et al., 2004; Yang and Wang, 2006).

Using AP-like stimulations, it has been shown that the action potential repolarization rate has a significant effect on the size of the calcium channel response (McCobb and Beam, 1991; Yang and Wang, 2006). Using modified AP-waveforms (Wu et al., 2004), and simulations (Bischofberger et al., 2002) it has been shown that small changes in the amplitude of the action potential have large effects on the size of the calcium current. However, the combination of how changes in AP amplitude and shape interact to affect calcium channel open probability and calcium currents has not been sufficiently addressed.

Here, we used the calyx of Held nerve terminal to study the presynaptic activity of voltage-gated calcium channels. This nerve terminal is of sufficient size to allow presynaptic patch-clamp recordings (Barnes-Davies and Forsythe, 1995; Borst et al., 1995). Apart from its size, the calyx of Held is a nerve terminal that contains voltage-gated calcium channels and vesicle release machinery that are found in presynaptic terminals throughout the brain (Schneggenburger and Forsythe, 2006; Borst and Soria van Hoeve, 2012; Körber and Kuner, 2016). The terminals primarily contain P/Q-type, along with N-type and a small population of R-type calcium channels (Wu et al., 1999; Ishikawa et al., 2005) that comprise the CaV2 group of voltage-gated calcium channels. These channels activate at approximately −40 mV, have fast activation (Borst and Sakmann, 1998) and have relatively slow inactivation rates relative to the duration of an action potential (Forsythe et al., 1998), such that minimal inactivation occurs even during repetitive stimulation (Wang and Kaczmarek, 1998).

To better understand the presynaptic calcium channel response to different types of brief AP-like stimuli, we tested short voltage-jumps and AP-like stimulation. We also studied the effects of varying the depolarization and repolarization rates using a series of AP-like stimuli with a fixed total duration at three different peak amplitudes. Finally, we used hyperpolarizing voltage jumps during the repolarization phase of AP-like stimuli to create a tail current which allowed us to measure the relative number of calcium channels that are active at different times during the repolarization. This measures the rate of calcium channel recruitment and shows the relative number of calcium channels that are active at different times during an AP-stimulus.

The results shown here demonstrate the relationship between the rising phase, decaying phase, and amplitude of the action potential on voltage-gated calcium channel activation and calcium entry. Specifically, by using fixed duration action potential-like stimuli, we have shown how the rise time, decay time, and amplitude interact to affect the calcium channel current.

## Materials and Methods

### Animals

CD1 albino mice at postnatal day 8 (PN8) to PN12 were used for the experiments in this manuscript. The mice were housed in a vivarium with procedures and facilities approved by the Association for Assessment and Accreditation of Laboratory Animal Care International. Utmost care was used in handling the animals, and the protocols used for handling and care of the mice were reviewed and approved by the Rutgers University Animal Care and Facilities Committee.

### Brain Slice Preparation

Mouse brains were sliced in the parasagittal orientation, on a precision vibratome (VT1200, Leica) at a speed of 0.01 mm/s through the medial nucleus of the trapezoid body. Slice thickness ranged from 150 to 170 μm. To minimize cellular damage during the dissection and slicing procedure, the brain was maintained at 1–2°C in a low calcium artificial cerebrospinal fluid (aCSF) solution consisting of (in mM): 125 NaCl, 25 NaHCO_3_, 25 glucose, 3 Myo-Inositol, 2.5 KCl, 2 Na-pyruvate, 1.25 NaH_2_PO_4_, 0.4% ascorbic acid, 3 MgCl_2_, and 0.1 CaCl_2_ at a pH of 7.3 when oxygenated with carbogen gas (95% oxygen, 5% carbon dioxide). To allow recovery from the slicing procedure, each slice was quickly transferred to a chamber maintained at 37°C for 30–45 min in a normal calcium aCSF solution with the composition listed above, except that 1 mM MgCl_2_ and 2 mM CaCl_2_ were used. This solution was also used as a recording solution. Following the 37°C incubation period, slices were stored at room temperature and bubbled with carbogen to maintain adequate oxygen levels. Experiments were done at room temperature, ~21°C. Brain slices were used for up to 6 h following the incubation time.

### Electrophysiology

An EPC10 USB double patch-clamp amplifier (HEKA, Harvard Bioscience) was used for data acquisition on a PC running PatchMaster software (HEKA, Harvard Bioscience). A sampling frequency of 200 kHz was used. Currents were filtered by a 4-pole Bessel filter at 3 kHz to remove residual high-frequency noise present in the recordings. The use of ramped waveform stimuli allowed us to approximate an action potential (AP) stimulus and systematically vary the depolarization and repolarization duration as well as the peak amplitude of the stimulus. Presynaptic calyx of Held nerve terminals were patched at proximity to the axonal heminode (Leão et al., 2005) to achieve the best quality voltage clamp of the terminal. To reduce the size of the axon, thus reducing potential space clamp errors, slices were made in the parasagittal orientation to limit axonal length. Presynaptic terminals were visually identified and patched using a BX-51 microscope (Olympus) with a 40x objective, using DIC imaging, IR light and a Dage IR-1000 analog camera. Fluorescent dye (0.10 mg/ml lucifer yellow) was initially added to the pipette solution to allow visual confirmation of the location of the recording concerning the heminode. Thick-walled borosilicate glass with a 2.0 mm outer diameter and an inner diameter of 1.16 mm (Sutter Instruments) was used to pull patch pipettes with a resistance of 2.5–5 MΩ for the presynaptic recordings and 4–10 MΩ for the postsynaptic paired postsynaptic recordings. Holding potentials were typically –80 mV for presynaptic terminals and –65 mV for postsynaptic cells. The series resistance (R_S_) for the voltage clamp recordings was within the range of 4–14 MΩ for the presynaptic terminal. Once a presynaptic whole-cell recording was achieved, gentle pressure or suction was applied if necessary to maximize the R_S_. To achieve adequate speed for these recordings, R_S_ compensation of 60–70% at 2 or 5 μs was applied. The fast Rs compensation speed was necessary to adequately record the responses to the brief stimuli used here. Although this greatly reduced the number of stable recordings, it produced an adjusted R_S_ of 2–6 MΩ, which was necessary for the rapid responses measured here. Recordings that did not meet these criteria were not considered for data analysis. A P/–4 leak protocol was applied to subtract passive leak and capacitive currents for the stimuli delivered to the presynaptic terminal. To isolate the presynaptic CaV2 channels, tetraethylammonium (TEA; 20 mM), tetrodotoxin (TTX; 1 μm), and 3, 4-diaminopyradine (DAP; 1 mM) were added to the aCSF recording solution to block voltage-gated sodium and potassium channels. For dual recordings, bicuculline (10 μm) and strychnine (1 μm) were added to the aCSF recording solution to block inhibitory inputs in the postsynaptic neuron. The pipette solution contained the following (in mM): 90 Cs-methanesulfonate, 20 CsCl, 10 TEA, 40 HEPES, 1 MgCl_2_, 0.5 EGTA, 5 phosphocreatine, 2 ATP, and 0.2 GTP, and was buffered to pH 7.3 using CsOH. The intracellular pipette solution for postsynaptic cells during dual recordings consisted of the following (in mM): 135 potassium gluconate, 20 KCl, 10 HEPES, 5 EGTA, 5 phosphocreatine, 2 ATP, and 0.2 GTP and was buffered to pH 7.3 using KOH. Junction potentials were not corrected. Reagents used for these experiments were purchased from Sigma Aldrich or Alfa Aesar, except for TTX which was purchased from Tocris Bioscience (R&D Systems).

### Data Analysis and Calculations

After meeting the above criteria, recordings of presynaptic calcium currents, and excitatory postsynaptic currents (EPSCs) for the dual recording experiments, were analyzed using Igor Pro (Wavemetrics) and Excel 2010 (Microsoft). Peak amplitude, current integral, and duration of the presynaptic calcium current and EPSC responses were measured manually or by using the Igor Pro unipolar peak/area detection procedure. The peak amplitudes measurements were made relative to the baseline level of the recording. Responses were baseline adjusted as necessary. A baseline threshold of −2*(peak to peak noise) was set and the first location where the signal crossed this baseline threshold was measured as the onset. The second location where the signal crossed the baseline threshold was measured as the end of the response. A separate peak detection threshold was set, typically at −100 pA, detected the peak amplitudes of our responses. Changes in the slope between consecutive data points determines the peak amplitude. All of the measurements were graphed onto the trace and visually inspected for accuracy. Traces that had irregularities due to random electrical noise, mechanical vibration, etc., were discarded from our analysis. To verify the accuracy of the measurements, we frequently measured traces manually and we did not find an appreciable difference between the inspected automated trace measurements and the measurements done by eye. Presynaptic calcium current integrals were measured from the onset of the inward calcium current and continued until the current returned to the baseline level. EPSC integrals were measured from the onset of the inward current to 5 ms after the onset of the inward current when the response had largely decayed. Statistical analysis was performed using Microsoft Excel. Data values are expressed as the mean ± the standard deviation (SD). Statistical significance was performed using Student’s *t*-test: two tails, two sample with equal variance (*p < 0.05; **p < 0.01; ***p < 0.005). Data were graphed using Microsoft Excel or Igor Pro. Data traces were arranged and displayed by graphing in Igor Pro.

Local calcium concentrations were calculated using the following formula (Dittman and Ryan, 2019):

(Ca^2+^ E_rev_ − Vm) γ_Ca_/(4π · F · D_Ca_ · r)

Where Ca^2+^ E_rev_ is the reversal potential for calcium (0.043 V); Vm is the membrane potential (V); γ_Ca_ is the CaV2 conductance (2.76 pS); *F* is the Faraday constant (96, 485.33 C/mol); D_Ca_ is the calcium diffusion rate (220 μm^2^/s), and *r* is the radius (nm).

## Results

### Area-Matched Stimuli Have Different Effects on Presynaptic Calcium Channel Activity

**Figure 1:**
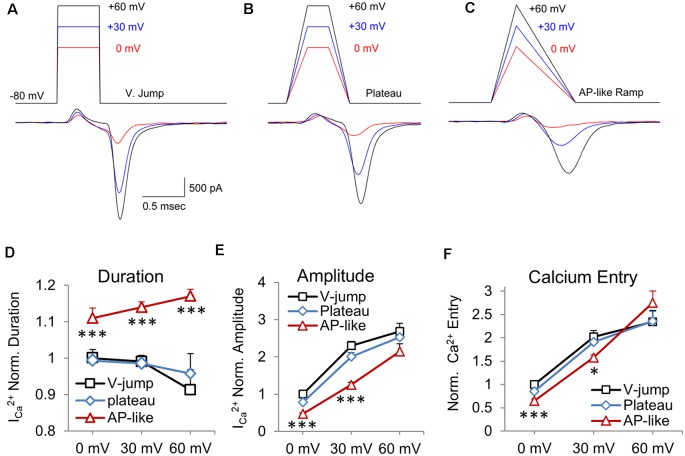
Area-matched stimuli demonstrate how the shape of the stimulus affects the presynaptic CaV2 channel response. Test pulses started at a holding potential of −80 mV and were depolarized to peak voltage values of 0 (red), +30 (blue), and +60 mV (black) and were constructed to have an equivalent area over a given peak voltage. The resulting calcium current appears below each pulse. **(A)** Voltage-jump stimuli maintained for 0.5 ms, with peak amplitudes of 0, 30 and 60 mV, followed by a return to holding potential of −80 mV. **(B)** Plateau wave stimuli, with matching stimulus areas to stimuli from panel **(A)**, consisting of a 0.25 ms depolarization to 0, +30, or +60 mV for 0.25 ms, followed by a 0.25 ms repolarization to baseline. **(C)** Ramped AP-like stimulus: 0.3 ms ramped depolarization and a 0.7 ms ramped repolarization. **(D–F)** Data is presented as the average of the presynaptic recordings (*n* = 5 cells). These are cells where all three stimuli were tested at the three different peak amplitudes for nine separate stimuli. **(D)** Normalized duration of the CaV2 response. The average value of the response to the voltage-jump stimulus was normalized to one. This average value was used to normalize the average responses to the AP-like and plateau stimuli. Differences in AP-like and V-jump stimulation at 0, 30 and 60 mV are statistically significant (*P* < 0.005). **(E)** Normalized peak amplitude of the CaV2 response corresponding to each of the test waveforms. Differences between AP-like and V-jump stimuli at 0 and 30 mV were statistically significant (*P* < 0.005). **(F)** Normalized calcium current integral (nA*ms) for the response to each stimulus. Differences between the AP-like and V-jump stimuli were statistically significant for the 0 mV values (*P* < 0.005) and the 30 mV values (*P* < 0.05). *p < 0.05; ***p < 0.005.

To determine if there are basic differences in calcium channel currents generated by voltage jump stimuli vs. stimuli with a ramped depolarization and repolarization, we first tested sets of stimuli with instant voltage jumps at three different peak amplitudes (Figure 1A, V. Jump). Next, we tested sets of stimuli with fast depolarizing and repolarizing phases (0.25 ms) with a peak plateau phase (Figure 1B, Plateau), and sets of stimuli with an AP-like depolarization (0.3 ms) and repolarization (0.7 ms) phase (Figure 1C, AP-like Ramp). Ramped stimuli have been used by several groups to mimic action potential stimuli (McCobb and Beam, 1991; Pattillo et al., 1999; Yang and Wang, 2006; Han et al., 2015; Montesinos et al., 2015). The rise and decay times closely match the rise time and decay of physiological action potentials in the calyx. Furthermore, the half-width duration of this stimulus is 0.5 ms, which is similar to the half-width duration for action potentials at the calyx of Held (Borst and Sakmann, 1998; Dodson et al., 2002). Each of the three different stimuli was tested at peak amplitudes of 0, 30 or 60 mV, and they have identical stimulus areas when compared at the same peak amplitude. Previous studies (Toth and Miller, 1995; Wu et al., 2004) and simulations (Graham and Redman, 1994; Bischofberger et al., 2002) have shown that the peak amplitude of an action potential waveform stimulus affects the size of the voltage-gated calcium current. Therefore, these experiments show how the response to voltage jump stimuli differ from the responses generated by stimuli with fast ramped voltage changes vs. responses from stimuli with AP-like depolarization and repolarization rates, across a range of peak amplitudes.

We quantified these data, by measuring the duration (Figure 1D), amplitude (Figure 1E) and total calcium entry (Figure 1F) for each of the three stimuli at the three different amplitudes tested. We show that the duration of inward calcium current decreases for the voltage jump stimulus as the stimulus amplitude increases from 0 to +60 mV (Figure 1D; *p* = 0.008). This is primarily due to a small amount of calcium entry at 0 and +30 mV that is not present at +60 mV. However, for the AP-like stimulus, the duration of calcium entry steadily increases as the amplitude of the stimulus voltage increases from 0 to +60 mV (*p* = 0.012, *n* = 5 cells). The duration of the CaV2 response to the 0 mV AP-like stimulus is ~10% longer than the responses to the voltage jump or plateau stimuli at 0 mV, and 30% longer by +60 mV. We also note that the duration of calcium entry in response to the AP-like stimulation shows a steady increase as the peak stimulus voltage increases (Figure 1D). Next, comparing the amplitude of the CaV2 responses, we find that the voltage-jump and plateau show a large, ~2-fold increase from 0 mV to 30 mV, but only a modest increase from 30 mV to 60 mV (Figure 1E). In contrast, the amplitude of the AP-like response is lower than the responses from the other two stimuli, but the AP-like response steadily increases as the peak stimulus voltage increases (Figure 1E). Interestingly, for calcium entry, the AP-like stimulus shows a larger range of responses as the peak amplitude of the stimulus increases (Figure 1E). Lastly, for total calcium entry, the AP-like stimulation is the least effective stimulus at 0 mV, but by 60 mV, the response to the AP-like stimulus matches or exceeds the response to the other two stimuli (Figure 1F). For total calcium entry, the amplitude of the stimulus has a greater effect on the total calcium entry than the stimulus shape does. We also note that similar to duration and amplitude of the calcium response, the total calcium entry generated by the AP-like stimulus shows a more linear increase for calcium entry at amplitudes from 0 to 60 mV, compared to the other two waveform stimuli we tested (Figure 1F). The finding that the AP-like stimulus produces a more linear increase in amplitude, duration, and total calcium influx is important because these factors affect the timing and peak Ca^2+^ concentration near the calcium channels and release machinery, and therefore affect the amount and timing of neurotransmitter release (Wang and Augustine, 2015).

### Small Changes in Presynaptic Calcium Influx Have Large Changes on Synaptic Release

**Figure 2:**
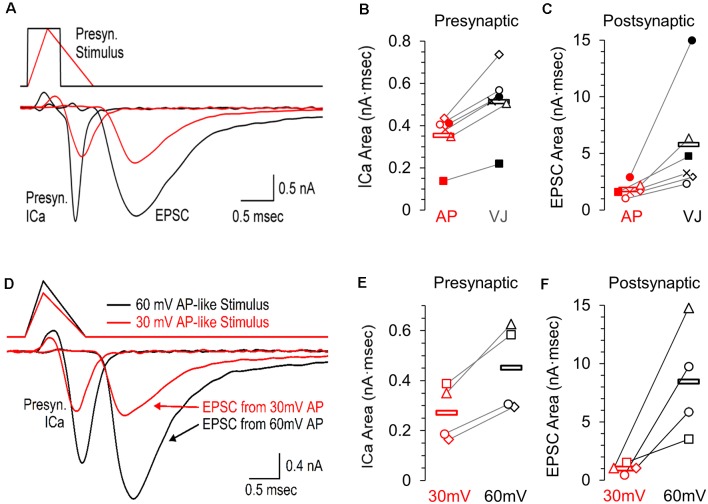
Presynaptic calcium channel activity and simultaneously recorded postsynaptic response to an AP-like stimulation, an area matched voltage-jump stimulation, and the effects of AP-like stimulation amplitude. **(A)** Simultaneous pre- and postsynaptic recordings using a presynaptic voltage-jump stimulus (black traces) or an AP-like stimulus (red traces) consisting of a 0.3 ms ramped depolarization and 0.7 ms ramped repolarization; both with a peak amplitude of +30 mV. These representative recordings from a single cell show the presynaptic calcium current, followed by the corresponding excitatory postsynaptic current (EPSC). **(B)** Individual values and average area of the presynaptic calcium current (*n* = 6 paired recordings) in response to the voltage jump (avg. 0.512 ± 0.068, black) or the AP-like stimulation (avg. 0.352 ± 0.044 SE, *n* = 6 paired recordings, red). **(C)** Individual values and average area of the EPSC (*n* = 6 paired recordings) in response to a presynaptic voltage jump stimulus (avg. 5.74 ± 1.93, black) or an AP-like stimulation (avg. 1.81 ± 0.26, red). **(D)** Simultaneous pre- and postsynaptic recordings using a presynaptic AP-like stimulus consisting of a 0.3 ms ramped depolarization and 0.7 ms ramped repolarization and a peak amplitude of 30 mV (red) or 60 mV (black). **(E)** Individual values and average area of the presynaptic calcium current (*n* = 4 paired recordings) in response to an AP-like stimulation with a peak amplitude of 30 mV (avg. 0.27 ± 0.05 SE, red) or 60 mV (0.45 ± 0.09, black). **(F)** Individual values and average area of the excitatory postsynaptic current (EPSC, *n* = 4 paired recordings) in response to an AP-like stimulation with a peak amplitude of 30 mV (avg. 1.01 ± 0.22 SE, red) or 60 mV (8.47 ± 246 SE, black).

It is difficult to fully predict how small changes in the presynaptic calcium current (Figures 1A–F) will affect synaptic transmission. Accordingly, we performed simultaneous presynaptic and postsynaptic recordings to show how the postsynaptic response to a presynaptic ramped AP-like stimulation (Figure 2A, red) differs from the response to a standard voltage jump (Figure 2A, black). The average presynaptic calcium influx for the response to the voltage jump stimulus (0.512 nA·ms ±0.068) was 1.4-fold greater than the response to the ramped AP-like stimulus (0.352 pA·ms ±0.045, Figures 2A,B; *n* = 6 paired recordings). In contrast, the area of the EPSC generated by the voltage jump stimulation (5.74 nA·ms ±1.93) was 3.2-fold higher than the EPSC response to the AP-like stimulation (1.81 nA·ms ±0.26, Figure 2C). It is well established that the postsynaptic response is highly sensitive to changes in intraterminal calcium (Matthews, 1996; Schneggenburger and Neher, 2000). Our results show how two stimuli with the same area affect the presynaptic calcium current which then scales up to create a much larger difference in the postsynaptic response.

To further demonstrate how the presynaptic calcium current from an action potential affects the postsynaptic response, we also tested paired recordings using presynaptic AP-waveforms with peak amplitudes of 30 mV and 60 mV (Figure 2D, *n* = 4 paired recordings). We find the presynaptic calcium current produced by a 60 mV AP-like stimulation (0.453 nA·ms ± 0.088) is 1.7-fold higher than the calcium current produced by a 30 mV AP-like stimulation (0.27 nA·ms ± 0.056, Figure 2E). On the postsynaptic side, the EPSC generated by a 60 mV presynaptic AP-like stimulation (8.47 nA·ms ± 2.46) was more than 8-fold higher than the response generated by a 30 mV presynaptic AP-waveform (1.01 nA·ms ± 0.22, Figure 2F). This agrees with other studies that have shown that the amplitude of the action potential can have large effects on neurotransmitter release (Wu et al., 2004; Zbili et al., 2016).

### Suprathreshold Stimulus Area and Total Presynaptic Calcium Influx

**Figure 3:**
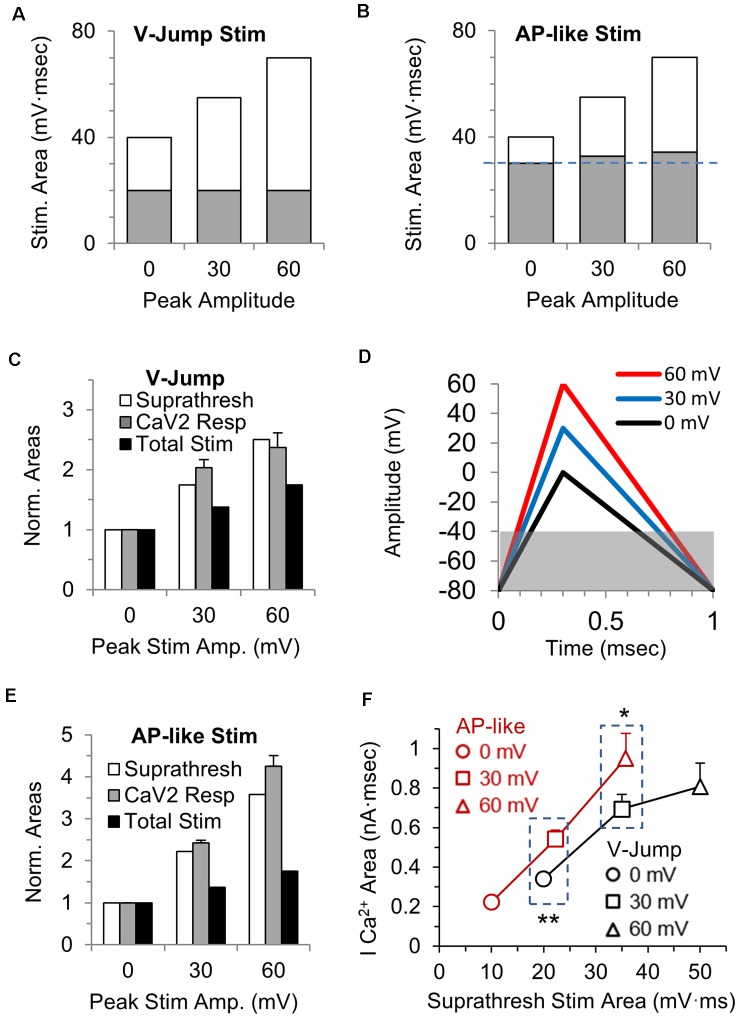
Stimulus area above −40 mV correlates with the area of the calcium channel response. **(A)** The total area of each voltage-jump stimulus, with the portion of the stimulus that is above the threshold for calcium channel activation shown in white, and the portion below threshold in gray. **(B)** The total area of each AP-like stimulus, with the portion of the stimulus above the threshold for calcium channel activation shown in white, and the portion below threshold in gray. The subthreshold area increases slightly (see panel **D**) as the AP-like stimulus amplitude increases (blue dashed line). **(C)** The suprathreshold stimulus area of each voltage-jump stimulus (white), the calcium current integral (gray) and the total stimulus area (black) at 30 and 60 mV were normalized to the value at 0 mV; *n* = 5 cells, from Figure 1F. The graph allows a comparison between the relative changes in the total or suprathreshold stimulus areas to the relative changes in calcium influx. **(D)** AP-like stimulus with peak amplitudes of 0 (black), 30 (blue) or 60 mV (red). Panels **(D–F)**: *n* = 5 cells, from Figure 1F. The gray box indicates the subthreshold portion of the stimulus, and how the subthreshold area differs between these stimuli. **(E)** The suprathreshold stimulus area of each AP-like stimulus (white), the calcium current integral (gray) and the total stimulus area (black) at 30 and 60 mV were normalized to the value at 0 mV. The graph allows a comparison between the relative changes in the total or suprathreshold stimulus areas to the relative changes in calcium influx. **(F)** Direct comparison of the calcium current areas and suprathreshold stimulus areas for voltage jump vs. AP-like stimulation with peaks amplitudes of 0, 30 and 60 mV. Note that the suprathreshold stimulus area are similar for a 0 mv voltage jump and a 30 mV AP-like stimulation (left box); and are also similar for a 30 mV voltage jump and a 60 mV AP-like stimulation (right box). At similar suprathreshold areas, the AP-like stimulation allows more calcium entry. *p < 0.05; **p < 0.01.

CaV2 channels in the calyx of Held nerve terminal begin to open at approximately −40 mV. For the voltage jump stimuli, the total subthreshold portion of the stimulus area is the same regardless of the stimulus amplitude (Figure 3A, gray portion), while the suprathreshold portion of the stimulus (white area) increases 2.5-fold over the three different amplitudes tested (Figure 3A, white portion). In contrast, for the AP-like stimulus, the area of the suprathreshold stimulus increases 3.5-fold over the three different amplitudes tested (Figure 3B, white portion). We reasoned that the total suprathreshold stimulus area should better estimate the size of the presynaptic calcium current than the total stimulus area would.

To determine if the suprathreshold stimulus area of the voltage-jumps correlates with the size of the presynaptic calcium current, we normalized the suprathreshold stimulus areas (Figure 3C, white), and normalized the integral of the calcium current responses (Figure 3C, gray) at 30 and 60 mV to the value at 0 mV. For comparison, the relative increase in the total stimulus area is also shown (Figure 3C, black). For the voltage-jump stimuli, the increase in total calcium influx at 30 and 60 mV is better predicted by the change in the suprathreshold stimulus area (Figure 3C, white) than it is by the change in the total stimulus area. This indicates that for brief voltage-jump stimuli, the suprathreshold stimulus area better predicts the amount of calcium influx over the range of stimulus amplitudes tested.

Next, we repeated this comparison for the AP-like stimuli. We first note that the suprathreshold stimulus area for the AP-like stimuli (Figure 3B, white) is a smaller percentage of the total stimulus area than it is for the voltage-jump stimuli (Figure 3A, white). Specifically, the subthreshold portion of the stimulus (Figure 3D, gray), is ≥1.5-fold larger in percentage of the total stimulus area than it is for the voltage-jump stimuli (Figure 3A, gray). We then calculated and normalized the suprathreshold stimulus areas (Figure 3E, white) and normalized the integral of the calcium current responses (Figure 3E, gray) to the AP-like stimuli at 30 and 60 mV to the values at 0 mV. The relative increase in the total stimulus area is also shown (Figure 3E, black). Similar to the simple voltage-jump stimulus, this graph also shows that the relative change in the suprathreshold stimulus area correlates better with the calcium influx than the relative change of the total stimulus area (Figure 3E). However, the suprathreshold stimulus appears to underestimate the influx of calcium for a 60 mV AP-like stimulation. This likely occurs due to additional differences in calcium entry that occur based on the shape of the stimulus.

To further determine how the suprathreshold portion of the voltage-jump and AP-like stimuli affect activation of calcium channels, we compared the absolute values of the calcium current areas and the suprathreshold stimulus areas for these two stimuli at peak amplitudes of 0, 30 and 60 mV (Figure 3F). Interestingly, the AP-like stimulation shows a steady increase in the total calcium entry for the suprathreshold stimulus area from 0 mV to 60 mV peak amplitudes. In contrast, for the voltage jump stimuli, the total calcium entry does not steadily increase for the suprathreshold stimulus area from 0 mV to 60 mV peak amplitudes. Furthermore, the AP-like stimulus produces a significantly larger influx of calcium compared to the voltage jump stimulus when the suprathreshold stimulus areas are similar at 20 mV·ms (*p* = 0.002) and 35 mV·ms (*p* = 0.01). Therefore, for the portion of the stimulus that activates voltage-gated calcium channels, the action potential stimulus is more efficient at producing a calcium influx compared to the voltage-jump stimulus. To further examine the activation and influx of calcium during AP-like stimulation and a full range of ramped stimulation waveforms, we tested a series of stimuli with identical areas and a 1 ms total duration but with different depolarization and repolarization duration as described in the next section.

### Calcium Influx Changes With the Duration of Depolarization vs. Repolarization and Peak Stimulus Amplitude

**Figure 4:**
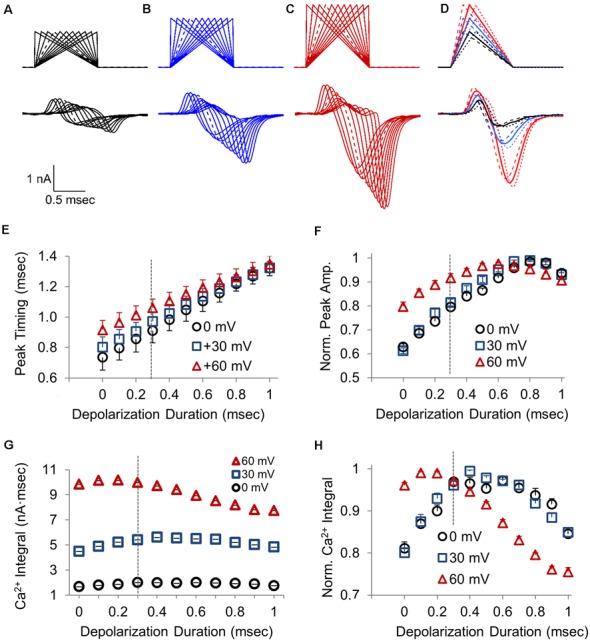
Changes in the calcium channel response resulting from differences in the duration of the depolarization and repolarization time for a 1 ms waveform stimulus. **(A–C)** Presynaptic CaV2 response to a series of 1 ms stimuli transitioning from a 0 ms depolarization followed by a 1 ms ramped repolarization to a 1 ms ramped depolarization followed by a 0 ms repolarization. Waveforms reached a peak voltage of 0 mV **(A)**, +30 mV **(B)**, or +60 mV **(C)**. Dashed lines are the stimulus and response to an AP-like stimulation with 0.3 ms depolarization and 0.7 ms repolarization. **(D)** Overlay of presynaptic CaV2 responses to stimuli with a 0.2 (dashed), 0.3 (bold), or 0.4 (dotted) ms ramped depolarization and the remaining ramped repolarization to produce a 1 ms stimulus, with a peak amplitude of 0 mV (black), +30 mV (blue), or +60 mV (red). **(E)** The time required for presynaptic CaV2 response to reach peak amplitude, with stimulus onset as *t* = 0; *n* = 10 cells. **(F)** Normalized peak amplitude for the presynaptic CaV2 current at different depolarization to repolarization times with the indicated peak stimulus amplitudes. Each data-trace at 0, 30 and 60 mV was normalized to the peak amplitude for that trace, and the normalized traces were then averaged (*n* = 10 cells) to compare the relative changes in peak amplitude at different depolarization durations. **(G)** Presynaptic calcium entry measured as the integral of the CaV2 current; *n* = 10 cells. **(H)** Normalized presynaptic calcium integral for the CaV2 response. Each data-trace at 0, 30 and 60 mV was normalized to the peak amplitude for that trace, and the normalized traces were then averaged (*n* = 10 cells) to compare the relative changes in peak amplitude at different depolarization durations.

The voltage-jump stimulus and the AP-like stimulus have the same area, but different durations and time course, which affects the total influx of calcium. To better understand how the rise and decay times affect calcium channel activity, we tested a range of area and duration-matched stimuli that all have the same suprathreshold stimulation area, but have different combinations of the rise and decay times. To determine how the amplitude of these stimuli affect the calcium channel response, we tested each stimulus at three different peak amplitudes. The stimuli range from a 0 ms rise (voltage jump) with a 1 ms decay to a 1 ms rise with a 0 ms decay, and every combination between this in 0.1 ms increments (Figures 4A–C, top). A total of eleven area matched stimuli with different rise and decay times were tested for three different stimulus amplitudes. We also hypothesized that the AP-like stimulus, with a 0.3 ms rise time and 0.7 ms decay time that closely matches the rising and falling phases of a physiological AP, would likely be an optimal stimulus to activate the CaV2-type voltage-gated calcium channels in this terminal (Figure 4D).

The representative overlay traces (Figures 4A–D) clearly show that the stimulus shape and amplitude have a large effect on the size of the presynaptic calcium current. The overlay of calcium currents at each potential shows how the CaV2 response onset correlates with the onset of the repolarization. This is seen in the graph of peak timing (Figure 4E) for the different depolarization durations. The onset rate of the calcium channel response (not shown) also increases linearly as the depolarization duration and the repolarization rate both increase. At each stimulus amplitude tested, the calcium current amplitude initially increases as the duration of the depolarization increases (Figure 4F). However, for all three amplitudes tested, the peak amplitude of the calcium channel response reaches a maximum value when the rise time is approximately 0.7 ms, and then slightly declines as the depolarization time reaches 1 ms with a repolarization of 0 ms which is a voltage jump (Figure 4F). This clearly shows how the duration time for the depolarization affects the amplitude and timing of the presynaptic calcium current and therefore the time required for synaptic transmission to occur and the size of the postsynaptic response.

Previous studies have shown that EPSC is better determined by the total presynaptic calcium entry than by the peak amplitude of the calcium channel response (Yang and Wang, 2006). Therefore we also measured the integral of each CaV2 response to determine the amount of calcium influx as a function of the depolarization to repolarization ratio and peak stimulus amplitude (Figure 4G). As expected, the amount of calcium entry changes with the different depolarization to repolarization ratios. Similar to what occurs for the peak amplitude, the amount of total calcium entry increases and then decreases as the depolarization time increases. However, the peak calcium entry occurs earlier, at a shorter depolarization duration of 0.4 ms or less (Figures 4G,H), compared to when the peak response occurs (Figure 4F). Interestingly, the decrease in calcium entry that occurs as the depolarization time increases is greatest for the stimuli with 60 mV peak amplitude, which is seen in the graph of the normalized traces (Figure 4H). The lower stimulation amplitudes, 0 and 30 mV, show maximum calcium entry when the depolarization reaches 0.4 ms. However, the 60 mV depolarization gives maximal calcium entry between 0.1 and 0.2 ms depolarization. Therefore there is a leftward shift for maximum calcium entry compared to the lower peak amplitudes tested. Since total calcium entry appears to be more influential than peak amplitude for releasing neurotransmitters, the faster action potential depolarization rates are more favorable for calcium entry and for minimizing synaptic delay. We also note that the normalized calcium entry for the three stimulus amplitudes converges at the AP-like stimulus that has a 0.3 ms depolarization and 0.7 ms repolarization (Figure 4H). This shows that the physiological timing of the action potential is optimized to produce a maximal response across a range of peak depolarization levels.

### Transient Upward Response Is Consistent With a Gating-Current

**Figure 5:**
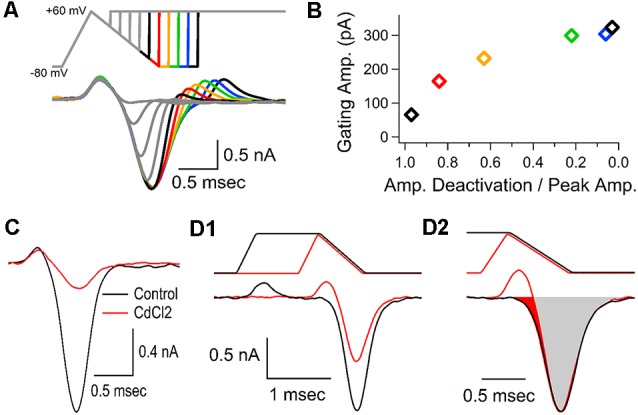
Gating current precedes the calcium channel response. **(A)** Representative traces, from a single cell, shown as an overlay of the calcium currents generated by 10 separate voltage-jumps to +60 mV, applied at regular intervals during an AP-like stimulus. Color traces following the peak of the calcium current show that the brief upward current re-appears as the stimulus returns to the holding potential, and the amplitude of the upward current increases as the amplitude of the remaining calcium current decreases. **(B)** Measurements from the data-trace in panel **(A)**. For each voltage jump, the amplitude of the calcium current at the onset of the voltage jump was divided by the peak amplitude of the calcium current and graphed with the amplitude of the second upward current (gating current). Measurements are color-coded to match the traces from panel **(A)**. **(C)** Cadmium (0.5 mM, 6 min) largely blocked the voltage-gated calcium channel response, but had little to no effect on the brief upward current. Traces shown are representative recordings from a single cell. **(D1)** To determine the calcium current in the absence of the brief upward current we compared the calcium current generated by an AP-like stimulus (black) to the response generated by an AP-like stimulus with a sustained depolarization that allows the upward current to return to baseline levels before the inward calcium current (red). Traces shown are representative recordings from a single cell. **(D2)** The response to the AP-like stimulation (**D1**, black) was scaled up to match the peak response from the prolonged AP-like stimulation. The filled red region indicates the portion of the calcium current that is affected by the upward gating current. In the representative cell shown here, the gating current obscured 5.4% (red area) of the inward calcium current.

A brief upward response appears immediately before the inward calcium current (Figures 1A–C, 2A, 4A–D). This brief upward phase is often present in calcium channel recordings (Borst et al., 1995; Forsythe et al., 1998; Bischofberger et al., 2002), but is less noticeable when longer stimuli are used to activate voltage-gated calcium channels. Some groups have proposed that the transient upward response is produced by a gating current (Toth and Miller, 1995; Borst and Sakmann, 1998). Other possibilities are that the transient upward response could be due to potential issues with the voltage-clamp recording, despite efforts to maximize the quality of the recordings (see “Materials and Methods” section). The brief initial upward response could also be caused by a brief outward flow of cations through voltage-gated calcium channels due to the small permeability of monovalent cations through calcium channels (Sather and McCleskey, 2003). Although intracellular calcium levels are too low to allow any appreciable flow of outward current, voltage-gated calcium channels could be slightly permeable to intracellular ions such as potassium ions, or cesium ions from the pipette solution (Fenwick et al., 1982). To address these possibilities, we used depolarizing voltage jumps applied at regular intervals during the repolarization phase of an action potential-like stimulation. If the upward response was due to the flow of ions through the calcium channels, we reasoned that depolarizing voltage jumps would reveal an outward flow of ions that should be larger when channels are already in an open state. If the brief upward response was an electrical artifact or due to a problem with the recording, the upward response should increase as the size of the depolarization increases. However, a second upward response is not visible when voltage-jumps are applied before the peak of the calcium current (Figure 5A, gray traces). Instead, we see a second upward response that gradually increases as the calcium channels deactivate during the repolarization, and this result is not consistent with an outward flow of ions or an electrical artifact. This second upward response reaches full recovery when the calcium current is near baseline levels and the calcium channels have deactivated (Figure 5A, green, blue and black traces). The finding that the size of the upward response increases with time after the AP-like stimulus ends (Figure 5A, traces after the red trace) demonstrates that the size of the upward response is inversely related to calcium channel activation. Accordingly, we measured the amplitude of the calcium current during the repolarization phase of the calcium current for each voltage jump and normalized this to the peak calcium current amplitude, then we measured the peak of the brief upward response for each voltage jump. The resulting graph (Figure 5B) shows that the upward response increases as the number of active calcium channels decreases. Interestingly, the recovery of the upward response is not linear but instead follows an exponential recovery similar to the exponential process of channel deactivation and recovery. Therefore the recovery of the brief upward response is most consistent with it being produced by gating currents that occur during voltage-gated calcium channel activation. This also indicates that the calcium channels that are activated by the AP-like stimulation have fully recovered by the time the calcium current returns to baseline levels.

To determine the contribution of the gating current to the calcium channel current, we attempted to isolate the gating current by applying cadmium to block the flow of calcium through voltage-gated calcium channels. By isolating the gating current, we could then subtract it from the calcium current to determine voltage-gated calcium channel response without the gating current. However, treatment with cadmium did not fully block the calcium channel current. We found that cadmium can block ~90% of the calcium current triggered by an AP-like stimulus, without affecting the gating current (Figure 5C). This shows that nearly eliminating the calcium flowing through the CaV2 channel does not affect the upward response, which is consistent with the upward response being due to gating currents, which still occur even though conduction through the pore is blocked. To fully determine how the gating current affects the inward calcium current that we are interested in, we tested an AP-like stimulus with a prolonged depolarization that allowed the gating current to return to baseline before the onset of the inward calcium current (Figure 5D1) and compared this to the response from a typical AP-like stimulation. Next, we scaled the size of the response from the AP-like stimulation to match the amplitude of the response generated by the AP-like stimulation with a prolonged depolarization (Figure 5D2). We then subtracted the areas of the two responses. This shows that the actual inward calcium current occurs slightly earlier than it appears to occur (Figure 5D2, red shaded area). The area of the subtracted current is an average of 5.2% (±2, *n* = 7 cells) larger than the area of the original trace. We conclude that this upward response is due to a gating current, which is present in all of our calcium current recordings and has only a small effect on our area measurements of the presynaptic calcium current.

### The Majority of Calcium Channel Activation Occurs During the Stimulus Repolarization

**Figure 6:**
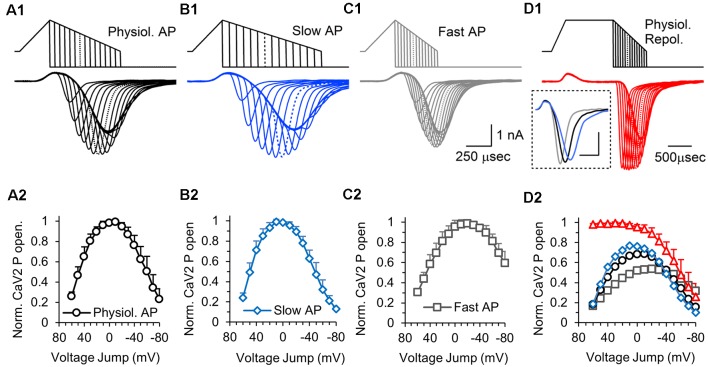
Effects of action potential repolarization duration on the number of presynaptic calcium channels that are activated at different times during the action potential. **(A1)** The top traces show a series of hyperpolarizing voltage-jumps, 15 in total, that were delivered at different times during the repolarization phase of a physiological AP-like stimulation. The bottom trace shows the overlaid presynaptic calcium channel responses to each voltage-jump. The peak amplitude measures the relative number of calcium channels that are active at each point during the repolarization. Dotted lines in the stimulus and response traces indicate the maximum response. Panels **(A1–D1)** show representative traces recorded from a single cell. **(A2)** Graph of the CaV2 open probability for a physiological AP stimulus, measured by the average CaV2 peak response to each voltage-jump, normalized to the maximum response for each cell. Panels **(A2–D2)**, *n* = 6 cells, with all 60 stimuli tested in each cell. The data points are the average of the normalized responses from each cell. **(B1)** Responses to hyperpolarizing voltage-jumps during an AP-like stimulation with a slow repolarization phase. **(B2)** Graph of the CaV2 open probability for a slow AP-like stimulus, measured by the average CaV2 peak response to each voltage-jump during, normalized to the maximum response (at +10 mV). **(C1)** Responses to hyperpolarizing voltage-jumps during an AP-like stimulation with a fast repolarization phase. **(C2)** Graph of the CaV2 open probability for a fast AP-like stimulus, measured by the average CaV2 peak response to each voltage-jump, normalized to the maximum response (at −20 mV). Inset shows **(D1)**. Prolonged depolarization to open all available presynaptic calcium channels followed by a series of hyperpolarizing voltage-jumps during an AP-like repolarization phase. Inset shows the full response to AP-like stimulation without a voltage jump for a normal (black), fast (blue) or slow (gray) repolarization (scale bar: 0.5 ms, 1 nA). **(D2)** Graph of the average CaV2 open probability for all four stimuli tested normalized to the responses to the long depolarization **(D1)** stimulus which should produce the maximum CaV2 open probability: long-depolarization (red triangles); physiological repolarization (black circles); slow repolarization (blue diamonds); fast repolarization (gray squares). The responses to each stimulus are normalized to the long depolarization maximum response for each cell, and the normalized data from each cell were averaged (red triangles).

The results from Figure 4 demonstrate how the repolarization time affects the calcium current. During the repolarization phase of an action potential stimulus, calcium channel activation and the electrical driving force for calcium entry are both changing, making it difficult to accurately determine the number of calcium channels that are active at specific points during the repolarization. To measure the relative amount of calcium channel activation during the repolarization phase of an AP-like stimulus, we used hyperpolarizing voltage jumps to generate a tail current response that allows us to compare responses at different times during the repolarization (Figures 6A1–D1). A total of 15 separate voltage jumps were applied at 10 mV increments, starting at +60 mV and ending at −80 mV. In these experiments, the hyperpolarizing voltage jump was to −150 mV to allow us to adequately measure the peak response at different points during the decay phase of the calcium current. We also tested voltage jumps to −120 mV (not shown) and the measurable currents gave similar normalized responses. Therefore, the voltage jump experiments provide a measurement of the relative number of active calcium channels at different times during the repolarization phase of the AP-like stimulation.

Using this series of hyperpolarizing voltage jumps, we tested calcium channel activation during a physiological AP-like stimulus with a 0.3 ms depolarization and a 0.7 ms repolarization time (Figure 6A1). A graph of the normalized calcium current amplitude at each voltage jump shows that the depolarization only opens ~25% of the channels that will be opened by the AP-like stimulation (Figure 6A2, 60 mV jump). The number of activated calcium channels continues to increase past 0 mV, and the peak amount of total presynaptic calcium channel activation occurs around −10 mV. Calcium channels can continue to be activated until the stimulus voltage is below the threshold for calcium channel activation, around −40 mV. Approximately 90% of the total calcium channels opened by the AP-like stimulation are open between 20 and −30 mV. By −60 mV, only 50% of the total calcium channels opened by the AP-like stimulation are open, but calcium entry through each open channel is now near maximal levels due to the electrical driving force. This provides a measurement of open channel probability for calcium channels throughout the repolarization phase of the AP. Also, although the number of active calcium channels significantly decreased as the AP repolarizes to baseline, the calcium channels that are open at the end of the action potential should have a bigger effect on neurotransmitter release at nearby vesicles due to the higher amount of calcium entering through each of these channels.

The duration of the repolarization phase has a large effect on the calcium current (McCobb and Beam, 1991; Sabatini and Regehr, 1997; Pattillo et al., 1999; Yang and Wang, 2006; Hoppa et al., 2014; Han et al., 2015). To determine how the duration of the repolarization phase affects the activation of calcium channels, we used a 0.3 ms depolarization and tested the calcium channel response to a slow repolarization (1 ms), or a fast repolarization (0.4 ms). For the slow repolarization (Figure 6B1), the peak activation level for calcium channels is left-shifted to a value of 10 mV (Figure 6B2). In contrast, the faster repolarization (Figure 6C1) has a peak that is right-shifted and occurs at −20 mV (Figure 6C2). Also, approximately 60% of the calcium channels that were activated by the stimulus with the fast repolarization are still active at the end of the stimulation compared to approximately 10% for the slower repolarization. Therefore, faster repolarization causes the maximal activation of calcium channels to occur later for the stimulus voltage and results in more calcium channels being open at the end of the stimulus while an increase in the duration of the repolarization has the opposite effect.

In the experiments described above, the calcium channels continued to open during the repolarization. To further study this, we reasoned that the voltage jumps would no longer show a steady increase in response if most or all of the available channels were open before the repolarization. Accordingly, we used the same 0.3 ms depolarization onset but then maintained the peak depolarization for 1 ms before transitioning to a 0.7 ms repolarization phase (Figure 6D1, top). Voltage jumps applied at the same 10 mV increments used previously, now show a much different response (Figures 6D1,D2). The prolonged depolarization serves to fully activate the voltage-gated calcium channels, which causes the response to start at a maximum amount and remain there for the first five to six voltage jumps. Then, by approximately 0 mV, the number of activated calcium channels begins to steadily decrease. This further demonstrates that the steady increase in response that is produced by hyperpolarizing voltage jumps during the repolarization phase of the AP-like stimuli (Figures 6A1–C1) is due to a steady increase in the number of active calcium channels during the stimulation. This experiment also allowed us to determine the relative number of calcium channels opened by the physiological, slow and fast AP-like stimuli. The four different stimuli were applied to each cell, which allowed us to normalize the responses from each of the AP-like stimuli to the maximal response produced by the 1 ms depolarization protocol (Figure 6D2). This shows that the stimulus with the slow repolarization activates ~75% of the maximum calcium channel amount, the physiological AP-like stimulation activates ~70%, and the stimulus with the fast repolarization activates only 50% of the maximal calcium channel activity. At the end of the repolarization, only ~15% of the calcium channels are active for the physiological AP-like stimulation, In summary, this graph directly shows how the timing of the repolarization phase affects the total number of calcium channels that are activated by AP-like stimuli. This graph also shows the percentage of activatable calcium channels that are active at different times during the repolarization.

### Effects of Action Potential Amplitude on the Number of Active Calcium Channels at Different times During the Action Potential

**Figure 7:**
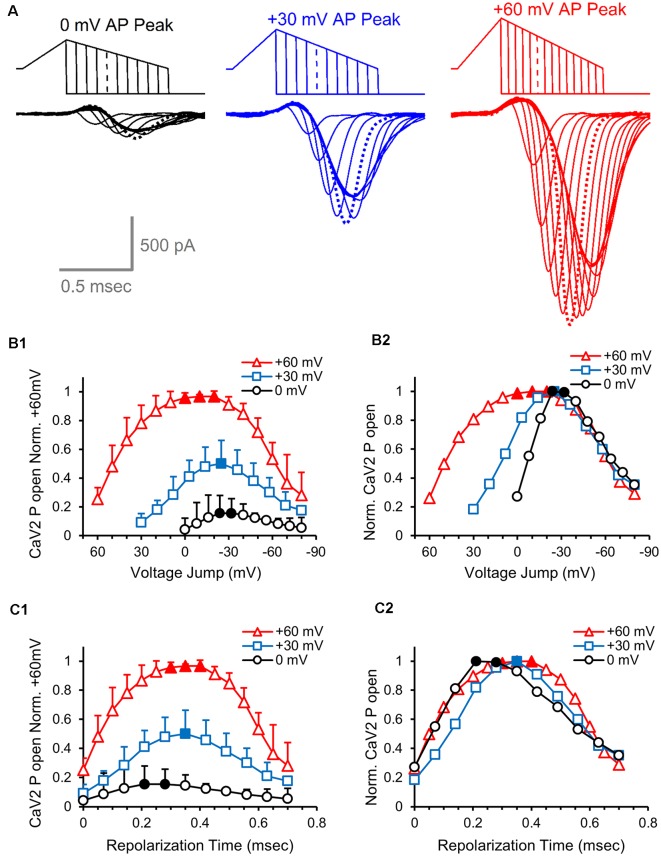
Effects of action potential amplitude on the number of active calcium channels at different times during the action potential. **(A)** Overlay of presynaptic CaV2 responses to hyperpolarizing voltage-jumps used to determine the relative number of open CaV2 channels at different points during the repolarization phase of an AP-like stimulus with peak amplitudes of 0 mV (black), 30 mV (blue) or 60 mV (red). Peak activation levels are indicated by dashed lines. **(B1)** Cav2 open probability measured by the peak amplitudes of the CaV2 responses to hyperpolarizing voltage-jumps. The values from each cell were normalized to the responses from the +60 mV stimulus, and the normalized data from each cell were averaged. The graph shows the relative differences in the number of active CaV2 channels at different repolarization membrane potentials for AP-like stimuli that peak at 0, 30 or 60 mV. Panels **(B1–C2)**, *n* = 5 cells. **(B2)** Relative CaV2 open probability produced by normalizing the values from panel **(B1)** to the peak response generated by each stimulus. **(C1)** Cav2 open probability measured by the peak amplitudes of the CaV2 responses to hyperpolarizing voltage-jumps. The data shown are from panel **(B1)**, with the values adjusted to show the response at specific repolarization times for the AP-like stimuli that peak at 0, 30 or 60 mV. The values from each cell were normalized to the responses from the +60 mV stimulus, and the normalized data from each cell were averaged. **(C2)** Relative CaV2 open probability produced by normalizing the values from panel **(C1)** to the peak response generated by each stimulus.

Next, we determined the presynaptic calcium channel activation during physiological AP-like stimuli with peaks at 0, 30 and 60 mV, again using a series hyperpolarizing voltage jumps (Figure 7A). As in the previous experiment, we measured the peak response at each voltage jump to determine the relative number of presynaptic calcium channels activated at different points during the repolarization phase. By normalizing all of the measurements to the peak response for the 60 mV stimulus, we find that a 60 mV AP-like stimulation opens ~2-fold more presynaptic calcium channels than a 30 mV AP-like stimulation, and 6–7-fold more calcium channels than a 0 mV AP-like stimulation (Figure 7B1). To better compare the presynaptic calcium channel at each of the three different amplitudes, we separately normalized the responses from each stimulus (Figure 7B2). This shows that the maximum activation level for all three stimuli occurs by −25 mV, followed by a very similar reduction of activated calcium channels. To compare the time course of calcium channel activation during the repolarization, we graphed the data as a function of repolarization time and normalized all responses to the peak calcium channel activation from the 60 mV AP-like stimulus (Figure 7C1). This shows the relative rate of recruitment for presynaptic calcium channel activation at the three different stimulus amplitudes. To directly compare the relative rates of recruitment, we separately normalized the responses to the peak amount of calcium channel activation for each stimulus (Figure 7C2). This shows that the rates of calcium channel recruitment and the reduction from peak calcium channel activation are similar for all three stimuli. This further indicates that amount of presynaptic calcium channel activation using AP-like stimuli scales with the total area of the stimulus used to activate the calcium channels (Figure 3E).

### Calcium Currents, the Open Probability of Calcium Channels, and the Steady Increase of Local Calcium Concentrations During the AP Repolarization

**Figure 8:**
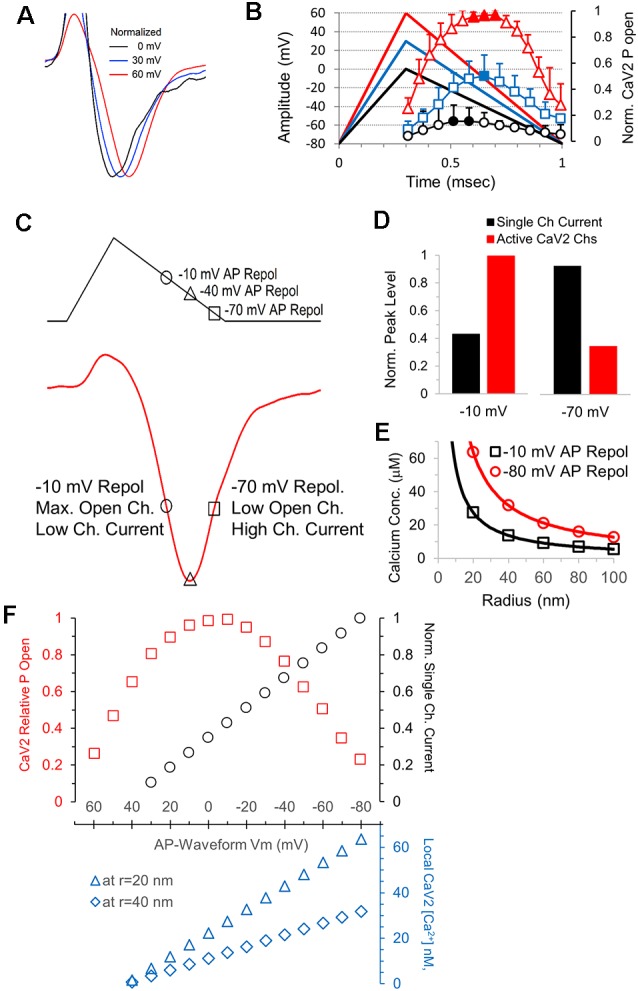
The electrical driving for calcium, not the number activated channels, determines the shape of the calcium channel response. **(A)** Responses shown in Figure 7 were normalized in amplitude to show how the time to reach peak amplitude increases as the amplitude of the stimulus voltage increases (60 mV, red; 30 mV, blue; and 0 mV, black. **(B)** Overlay of the AP-like stimuli and the CaV2 open probability data from Figure 7C shows the relationship between the stimulus time and the amount of CaV2 activation. **(C)** The AP-like stimulation and corresponding CaV2 current to show the relationship between the repolarization voltage at −10, −40 and −70 mV and the calcium current. The current at −10 and −70 mV are similar in this example, but the number of open calcium channels and the single-channel current differ greatly. **(D)** Graphs of the relative single-channel current (black) and CaV2 open probability (red) at repolarization values of −10 and −70 mV. The increased single-channel conductance at −70 mV offsets the reduction in the number of active calcium channels. **(E)** The single-channel current at when the AP repolarization is at −10 mV (black) and −80 mV (red) was used to calculate the local calcium concentration at individual CaV2 channels, at distances of 20–100 nm (radius). Channels that are still active at −80 mV have >2-fold higher local calcium concentrations than the channels that are active at −10 mV. **(F)** Graph of the relative CaV2 open probability, single-channel current, and local calcium concentrations during the AP-like repolarization from 60 to −80 mV. Local CaV2 calcium concentrations at the different membrane potentials are shown for a distance of 20 nm (blue triangles) and 40 nm (blue diamonds).

Consistent with the time to reach maximum calcium channel activation during the AP repolarization (Figures 7C1,C2), the time to reach peak current amplitude for CaV2 responses also increases as the amplitude of the AP stimulus increase. The peak amplitudes of the calcium channel response to AP-like stimuli with peak amplitudes of 0, 30 and 60 mV show a steady increase in the time to reach peak amplitude (Figure 8A). This is likely due to differences in the repolarization rates for the three different stimuli, differences in the timing of maximum calcium channel activation, and the change in single calcium channel conductance throughout the stimuli. The repolarization rate of the 0, 30, and 60 mV stimuli are 114.3, 157.1 and 200 mV/ms, respectively. To allow comparison between the relationship between peak calcium channel activation and the repolarization slope and timing, we graphed the 0, 30 and 60 mV AP-waveforms with the relative number of active calcium channels (Figure 8B). We then measured a series of normalized representative traces (not shown) and found that the AP-like stimulation produced a peak calcium current at 0.6 ms for 0 mV stimulation, 0.71 ms for the 30 mV stimulation, and 0.78 for the +60 mV stimulation. These values indicate that the peak calcium current occurs slightly after the peak in the open probability of calcium channels. This led us to further investigate the relationship between the open probability of calcium channels and the single-channel current during the AP repolarization.

We first looked at the peak calcium current and values shortly before and after the peak (Figure 8C). The peak in the open probability of calcium channels occurs near −10 mV during the repolarization, and the corresponding value in the rising phase of the calcium current is shown. The peak in the calcium current occurs near -40 mV during the repolarization. Lastly, we show the point during the calcium current decay that occurs at −70 mV during the repolarization. The single-channel calcium current is relatively low at −10 mV and relatively high at −70 mV, and we note that the calcium current is similar at both points (Figure 8C). To further illustrate this, we directly compared the calculated single-channel calcium current and the relative open channel probability at −10 mV and −70 mV (Figure 8D). This clearly shows how the calcium channels active at the end of the AP will allow a greater amount of calcium entry. To show how differences in calcium entry affect the local calcium concentration, we calculated the expected calcium concentration in a radius of 20–100 nm from an open calcium channel (Figure 8E). This indicates that calcium channels that are active at the end of the action potential produce a local calcium concentration that is over 2-fold higher than channels that are active at −10 mV, which corresponds to the midpoint of the repolarization phase and the point of maximum open probability for the calcium channels. Therefore, calcium channels that are active at the end of the action potential will have a higher local calcium concentration, and active zones containing these calcium channels should have a higher probability of release.

During an AP stimulation, the calcium channel open probability and the local calcium concentration both change throughout the repolarization. In Figure 8F, we show the number of active channels (red squares) and the calculated single-channel conductance (black circles) throughout the AP repolarization at intervals of 10 mV. The local calcium concentration at a single calcium channel is proportional to the single-channel conductance, and the local calcium concentration at a radius of 20 nm and 40 nm is shown in the lower graph (Figure 8F, blue). Therefore, the increase and decrease in the open probability of calcium channels and the steady increase in local calcium concentration during the AP repolarization are important in determining the probability of vesicle release in response to an AP.

## Discussion

Calcium channels are typically studied using depolarizing voltage jumps of several milliseconds or longer to allow the calcium current to reach a maximum level of activation. The repolarization jump back to baseline produces a large inward tail current response due to the increased electrical driving force for calcium (Eckert and Ewald, 1983). The tail current rapidly decays as calcium channel deactivation occurs in response to the change in membrane potential. For the 0.5 ms voltage jump used here, the visible calcium channel current is entirely due to a tail current. In contrast, during the repolarization phase of the AP-like stimulation, the electrical driving force for calcium progressively increases and calcium channels continue to activate during a large portion of the repolarization phase. Therefore, the speed and duration of the repolarization affect the number of open calcium channels and the electrical driving force for calcium entry.

### Fundamental Differences in the CaV2 Response to a Voltage-Jump vs. AP-Like Stimulation

Using a standard voltage-jump stimulation and a ramped AP-like stimulation with equal stimulus areas, we have shown how an AP-like stimulation produces a broader calcium current response that steadily increases with the amplitude of the stimulus compared to amplitude matched voltage-jump stimuli (Figure 1). Since the stimuli at each peak amplitude were identical in area, the differences demonstrate how the shape and duration of the stimulus affect the amount of calcium that enters the nerve terminal. The release of neurotransmitters in response to calcium entry through presynaptic voltage-gated calcium channels is highly sensitive to small changes in the calcium current. Using simultaneous pre- and postsynaptic recordings, we show that the AP-like stimulation has an ~30% smaller presynaptic calcium current compared to an area match voltage-jump stimulation. This decreased calcium current results in an ~80% reduction in the total area of the postsynaptic response. These results demonstrate how the shape of the stimulus affects the calcium current, and the subsequent postsynaptic response (Figure 2).

Action potential stimuli are for a brief duration, with a rapid depolarization followed by a slightly slower repolarization phase. These factors affect the activation of the CaV2 channels in several ways. First, for the ramped AP-like stimuli, the duration of the subthreshold portion of the stimulus is relatively brief and does not appear to affect the activation of the CaV2 channels. Therefore, the amount of CaV2 activation appears to be proportional to the suprathreshold stimulus area (Figures 3C,E), which is the total portion of the stimulus that is above −40 mV (Figures 3A,D). Second, increasing the pulse duration reveals additional calcium channel activation (Figures 5D1, 6D1,D2) which demonstrates that the duration of the AP-like stimulus is not sufficient to activate all of the calcium channels (Borst and Sakmann, 1998). Third, in response to AP-like stimuli, the majority of CaV2 activation occurs during the repolarization due to the brief duration of the depolarization phase. For the population of channels that will open in response to the stimulus, only ~25% of the channels will activate during the depolarization (Figure 7). In summary, the shape and duration of AP-like stimuli have distinct effects on calcium channel activation and the electrical driving forces for calcium entry.

We initially focused on the differences between voltage-jump stimuli and a ramped AP-like stimulation (Figures 1–3). Although these two stimuli had the same stimulus area, they differ in their fundamental shape and they differ two-fold in their duration. Accordingly, to focus specifically on how the shape of the action potential affects the calcium current, we used a fixed 1 ms duration and tested a series of ramped stimuli with different depolarization and repolarization durations (Figure 4). These experiments clearly show how the stimulus shape, or depolarization to repolarization ratio, affect the presynaptic CaV2 current. We find that the peak amplitude of the CaV2 response steadily increases until the depolarization duration reaches 0.7 ms, then it decreases slightly (Figure 4F). Combined with the data showing that the total calcium entry also decreases with longer depolarization duration (Figures 4G,H), the findings show that a higher depolarization to repolarization ratio decreases the amplitude and amount of calcium influx. Furthermore, the time between the stimulus and the peak amplitude of the CaV2 current steadily increases as the duration of the depolarization increases. This delay in the calcium channel response would act to delay synaptic transmission. In summary, the speed and magnitude of synaptic transmission are more favorable when the depolarization is fast.

In our recordings, the transient upward response that occurs before the inward calcium current is typically hundreds of picoamps in amplitude (Figure 5), similar to the estimated amplitude stated by earlier studies (Borst and Sakmann, 1998). The amplitude of this transient upward response increases with stimuli that produce a larger calcium current (Figures 4A–C), but is not decreased when the calcium current is blocked (Figure 5C). It is also absent during voltage jumps when calcium channels are already activated, and it steadily recovers as the calcium channels deactivate (Figures 5A,B). This activity is consistent with the transient upward response being produced by a gating current. Since the gating current from voltage-gated sodium channels is not blocked by TTX (Armstrong and Bezanilla, 1974), sodium channel gating is likely to also contribute to the gating current in our recordings. It should also be noted that our recording pipette was typically placed at, or very close to the heminode of the calyx of Held, and sodium channel density is high in this region (Leão et al., 2005). In summary, our results strongly suggest that the upward response is due to a gating current produced by voltage-gated calcium and sodium channels, but this gating current does not significantly obscure the inward calcium current. Therefore, the upward response is not significantly interfering with our measurements.

### CaV2 Activation From the Onset to the End of the AP Repolarization

To better understand how calcium channel activation occurs during the AP, we used hyperpolarizing voltage jumps to normalize the response at different times during the repolarization. This very useful technique has previously been used in somatic recordings of calcium channel activity (Pattillo et al., 1999). This allowed us to measure the relative amount of presynaptic calcium channel activity at different times during the action potential. Although the depolarization phase of the action potential begins the activation of voltage-gated calcium channels, the majority of calcium channel activation occurs during the repolarization. It is important to remember that the electrical driving force for calcium entry steadily decreases during the action potential depolarization, and then steadily increases during the repolarization. Therefore, during the repolarization phase, channels are continuing to open and the driving force for calcium entry is steadily increasing. Therefore, the repolarization phase largely controls the duration and amount of calcium entering the nerve terminal in response to an action potential.

### CaV2 Channels Active at the End of the AP Will Have a Larger Influx of Calcium Per Channel

The peak of the calcium current corresponds to approximately −50 mV during the repolarization of the AP-like stimulation (Figure 6A1). The finding that approximately half of the calcium channels that open in response to the action potential are still open at the peak of the calcium channel response (Figure 6A2) has important implications for synaptic release. After the peak response, the net calcium current is decreasing due to the deactivation of the calcium channels, but active calcium channels will have a larger current due to the increased electrical driving force for calcium entry (Figures 8C,D). Approximately one-quarter of the calcium channels opened by the action potential (23.2% ± 10.3, *n* = 6 cells) are still open when the action potential returns to baseline (Figure 6A2). Calcium entry through these channels will create a larger local calcium concentration (Figures 8E,F) which should act to increase the probability of triggering the fusion of vesicles that are further from the active calcium channels (Eggermann et al., 2012).

### CaV2 Channels Mediate Vesicle Fusion at Many Presynaptic Terminals in the Brain

Many presynaptic terminals are thought to contain CaV2-type calcium channels. However, alternative splicing or post-translational mechanisms including association with auxiliary subunits affect the kinetics and activity of the calcium channels in nerve terminals (Lipscombe et al., 2013). Accordingly, the CaV2 calcium channels at the calyx of Held are likely optimized to respond to the kinetics of the action potential at the calyx of Held and appear to be optimized to allow high-frequency activity. Concerning the effects of the action potential, a small increase in the duration of the repolarization phase of the action potential has significant effects on synaptic transmission through a slight increase in calcium influx. Similarly, a change in the peak amplitude of the action potential by as little as 5–10 millivolts has a slight effect on calcium entry and a substantial effect on neurotransmitter release (Wu et al., 2004). Importantly, the amplitude and shape of the action potential are not fixed values, but can be modulated or can change with activity. For example, due to the inactivation of sodium channels (Milescu et al., 2010) and potassium channels (Dodson and Forsythe, 2004), repetitive firing can broaden the action potential and decrease the peak amplitude (Borst and Sakmann, 1999). Also, the shape of the action potential can be modulated in the axon by activation of ionotropic and metabotropic receptors (Sasaki et al., 2011), and in nerve terminals by neurotransmitter (Stephens and Mochida, 2005).

In summary, we show that the voltage-gated calcium channel response to a ramped AP-like stimulus is not a simple tail current response. We demonstrate that altering the ratio of depolarization to repolarization for a fixed duration stimulus can produce a 20–25% change in the influx of calcium. Furthermore, changing the stimulus amplitude has a larger effect on calcium influx than changes to the repolarization and depolarization rates have. This is at least partially due to an increase in the overall stimulus area that occurs as the peak amplitude of the stimulus increases. Measuring total calcium channel activity, we find that the depolarization phase of an AP-like stimulus opens approximately 20% of the available calcium channels, while a maximum activation of ~70% of the available calcium channels occurs when the repolarization reaches ~0 mV (Figure 6D2). It is not possible to determine from our data if there is a population of presynaptic calcium channels that require a larger or longer depolarization to activate, or if the remaining channels not opened by a single action potential could be opened by the next action potential. We also note that the calcium channels that are still active at the end of the action potential (Figures 6A2,D2), will have an approximately three-fold higher influx of calcium compared to the calcium influx when the action potential repolarization is at 0 mV. Therefore, the interactions between the open probability of calcium channels and the steady increase in single-channel conductance that occur during the AP repolarization need to be carefully considered to understand how APs trigger neurotransmitter release. Also, calcium entry into the presynaptic terminal can modulate calcium channel activity (Mochida et al., 2008; Nanou et al., 2016) and trigger additional effects such as post-translational modulation, and recently discovered local protein synthesis in presynaptic terminals (Younts et al., 2016; Scarnati et al., 2018; Hafner et al., 2019) are likely to be affected by presynaptic activity which could be triggered by calcium influx. Therefore, the shape of the action potential, and the calcium channel response can have complex effects on synaptic transmission.

## Data Availability Statement

The datasets generated for this study are available on request to the corresponding author.

## Ethics Statement

The animal study was reviewed and approved by the Rutgers University Institutional Animal Care and Use Committee (IACUC).

## Author Contributions

All authors designed the experiments, analyzed data, and wrote the manuscript. MS and SC performed the experiments.

## Conflict of Interest

The authors declare that the research was conducted in the absence of any commercial or financial relationships that could be construed as a potential conflict of interest.
